# Desmosine as a biomarker for the emergent properties of pulmonary emphysema

**DOI:** 10.3389/fmed.2023.1322283

**Published:** 2023-12-18

**Authors:** Jerome Cantor

**Affiliations:** College of Pharmacy and Health Sciences, St John’s University, Queens, NY, United States

**Keywords:** elastin, elastic fibers, desmosine, pulmonary emphysema, emergent phenomena

## Abstract

Developing an effective treatment for pulmonary emphysema will require a better understanding of the molecular changes responsible for distention and rupture of alveolar walls. A potentially useful approach to studying this process involves the concept of emergence in which interactions at different levels of scale induce a phase transition comprising a spontaneous reorganization of chemical and physical systems. Recent studies in our laboratory provide evidence of this phenomenon in pulmonary emphysema by relating the emergence of airspace enlargement to the release of elastin-specific desmosine and isodesmosine (DID) crosslinks from damaged elastic fibers. When the mean alveolar diameter exceeded 400 μm, the level of peptide-free DID in human lungs was greatly increased, reflecting rapid acceleration of elastin breakdown, alveolar wall rupture, and a phase transition to an active disease state that is less responsive to treatment. Based on this finding, it is hypothesized that free DID in urine and other body fluids may serve as a biomarker for early detection of airspace enlargement, thereby facilitating timely therapeutic intervention and reducing the risk of respiratory failure.

## Introduction

The pathogenesis of pulmonary emphysema may involve multiple mechanisms acting on a number of different tissue components. Nevertheless, a primary feature of the disease is damage to the lung elastic fiber network (1, 2). Neutrophils and macrophages recruited to the lung by tobacco smoke and other toxic substances release enzymes and oxidants that degrade elastic fibers, thereby disrupting the mechanical forces responsible for expansion and contraction of alveoli (3, 4). The continued degradation of these fibers results in distension and rupture of alveolar walls, reducing lung surface area and impairing gas exchange.

While the relationship between excess protease activity and alveolar wall injury plays a central role in pulmonary emphysema, other mechanisms may be more directly responsible for the morphologic abnormalities associated with the disease. Changes in the distribution of mechanical forces may be necessary for converting proteolytic injury into airspace enlargement (5, 6). This hypothesis is supported by in-silico studies showing that local variations in alveolar wall elasticity evolve into global morphological alterations that resemble those seen in pulmonary emphysema (7).

This finding is consistent with the principle of emergence in which complex interactions at different levels of scale produce spontaneous reorganization of chemical and physical systems (8, 9). An example of emergent phenomena is an epidemic, in which the transmission of the disease vector depends on the unpredictable interaction of a variety of factors, such as population density and mobility (10). The incidence of infection may therefore remain uncertain until it involves a relatively large population.

The progression of pulmonary emphysema may incorporate analogous mechanisms, where a number of indeterminate events regulate the transition from normal to enlarged airspaces. The remodeling of the lung cannot be predicted by analyzing individual components such as elastase activity or antiprotease levels but may instead require identification of specific patterns of molecular and macroscopic behavior that are part of a self-organizing process at multiple levels of scale (7).

### A percolation model of structural changes in elastic fibers

The destruction of the elastic fiber network in pulmonary emphysema may initially involve only scattered foci within the lung parenchyma, with minimal architectural modifications. However, as injury to these fibers progresses, increasingly uneven transmission of forces through the lung results in alveolar wall rupture, a significant decrease in lung surface area, and markedly reduced gas exchange. These structural alterations may be modeled using percolation theory, which involves analyzing the movement of fluid-like materials through a network of channels (11). This concept provides a useful approach to investigating emergent phenomena, where small-scale events evolve into systemic transformations.

The effect of elastic fiber changes on lung mechanics may be modeled by using a specific percolation system known as a random resistor network in which conducting bonds are indiscriminately disconnected, thereby increasing the resistance to flow (12). The network is composed of two different interconnecting units (K1 and K2), respectively corresponding to either intact or fragmented fibers, where the K2 units are associated with resistance to the transmission of force (13). The 2 units are randomly arranged within a three-dimensional lattice, and their relative proportion determines how mechanical forces percolate through the lung. When there are few K2 units, the forces are diffusely spread through the stronger K1 units, minimizing disruption of lung architecture. However, as the proportion of K2 units increases (corresponding to elastic fiber injury), these forces become progressively concentrated in the remaining K1 units. The enhanced mechanical strain on these units accelerates their breakdown into K2 units, which is associated with a phase transition involving a loss of lung elastic recoil, hyperinflation of alveoli, and rupture of alveolar walls. At higher levels of scale, these changes are accompanied by an increase in pulmonary compliance and a reduction in gas exchange that can lead to respiratory failure.

Due to the similarity of this model to the flow of electricity through a matrix of conducting bonds, the formula for current density may be used to describe the transmission of forces through the K1 and K2 units, as follows:


$$j=\rho_{1}v_{1}+\rho_{2}v_{2}$$

where j represents current density, and $\rho_{1}v_{1}$ and $\rho_{2}v_{2}$ are the charge density ( ρ ) and velocity ( v ) of the strongly and weakly conducting bonds, respectively (14).

The flow of electrical current through these bonds is analogous to the transmission of mechanical force in our model, where $\rho_{1}v_{1}$ and $\rho_{2}v_{2}$ correspond to the force density in K1 and K2 units, respectively.

Despite the limitations of the analogy imposed by physical differences between mechanical force and electrical current, the effects of structural changes in elastic fibers may nevertheless be described in terms of the parameters responsible for conductance, according to the following formula:


$$G=\frac{A}{\mathrm{\rho l}}$$

where G is conductance, and A , l , and ρ are the area, length, and resistivity of the conducting material, respectively.

**Figure 1:**
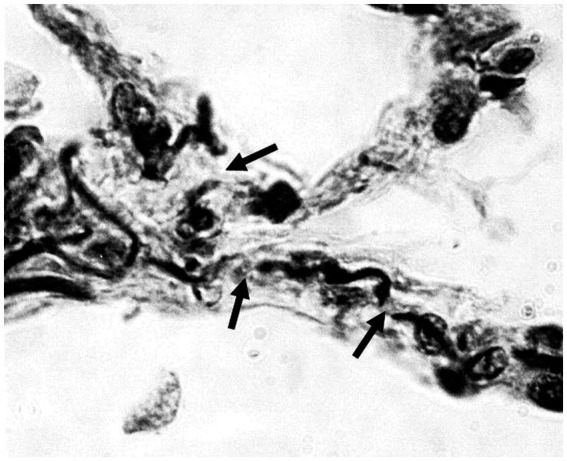
Photomicrograph of human emphysematous lung showing fragmentation of elastic fibers (arrows). Orcein stain; 1,000x magnification.

Applying this relationship to the lung, fragmentation and distention of the elastic fiber network, involving a loss of area and an increase in linear dimension, would disrupt the transmission of mechanical forces associated with breathing and increase alveolar wall strain (Figure 1).

### Elastin crosslinks as a biomarker for alveolar wall injury

The mechanical properties of elastic fibers depend on their ability to store and release energy by assuming different states of entropy (15). Distention of these fibers during inspiration is associated with a more ordered arrangement that decreases the level of entropy, while expiration reverses this process, producing the mechanical recoil needed to expel air from the lung. The changes in entropy are related to the properties of the core elastin protein which contains coiled, hydrophobic domains that interact with surrounding water molecules (16).

The structural integrity of elastin is dependent on desmosine and isodesmosine (DID) crosslinks, synthesized by the condensation of lysine residues within adjacent peptide chains (17). The importance of crosslinking was demonstrated in a study using beta-aminopropionitrile, an elastin and collagen crosslink inhibitor, to modify cadmium chloride-induced lung injury (18). Treatment with this agent produced pulmonary emphysema instead of interstitial fibrosis.

As a result of their unique presence in elastin and the very low turnover of elastic fibers in normal tissues, DID may potentially serve as a biomarker for lung injury associated with pulmonary emphysema. Increased amounts of DID are seen in blood, urine, and sputum from patients with pulmonary emphysema, and plasma levels of DID correlate with the loss of lung mass, as measured by high-resolution CT imaging (19–25). Nevertheless, one of the limitations of using blood or urine levels of DID is the release of these crosslinks from sites other than the lung. The coexistence of diseases such as arteriosclerosis or osteoarthritis that involve elastic fiber damage could obscure the increased levels of these crosslinks derived from the lungs. Plasma DID levels were shown to be more closely related to cardiovascular disease than lung function, and factors such as age, gender, body mass index, and smoking may affect the sensitivity and specificity of the biomarker for alveolar wall injury (26, 27).

**Figure 2:**
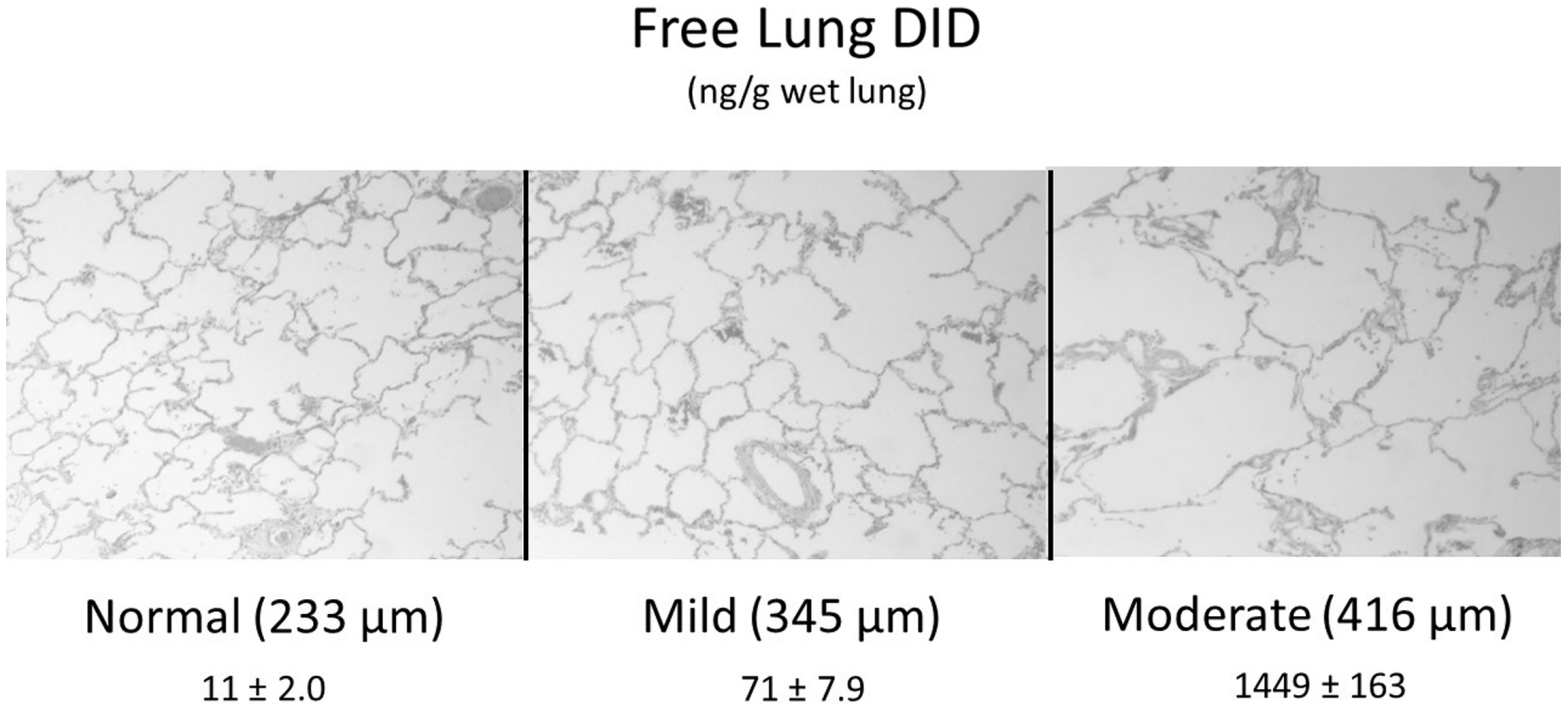
Elastin degradation in human pulmonary emphysema, as measured by the level of free lung DID, is greatly accelerated when mean airspace diameter (shown in parentheses) exceeds 400 μm (25). DID values are mean ± standard error of the mean. *N* = 10 for each group.

To address this problem, our laboratory performed measurements of peptide-free DID in the lungs of hamsters with pulmonary emphysema induced by treatment with both cigarette smoke and lipopolysaccharide (28). The results showed a significant correlation between free lung DID and increasing airspace size, supporting the concept that these crosslinks may serve as a biomarker for airspace enlargement. This study was followed by measurements of free lung DID in both normal and emphysematous postmortem human lungs, which showed that elastin breakdown was greatly accelerated when mean airspace diameter exceeded 400 μm (Figure 2) (29). The density of DID in lung tissue sections also increased markedly beyond 300 μm and leveled off at 400 μm.

These results suggest that the initial stages of alveolar wall injury are characterized by a balance between elastic fiber injury and repair in which greater crosslink density reflects enhanced elastin synthesis. However, when alveolar diameter exceeds 400 μm, the repair process undergoes a decompensatory phase involving a marked loss of DID and fragmentation of elastic fibers. The rapid acceleration of elastin breakdown with increasing alveolar wall distention supports the hypothesis that pulmonary emphysema is an emergent phenomenon involving a phase transition to an active disease state that is less amenable to therapeutic intervention.

### Diagnostic and therapeutic implications

The development of therapeutic agents for pulmonary emphysema may depend on the availability of a mechanism for real-time assessment of their efficacy. Currently, the only recognized clinical trial endpoints are pulmonary function studies, which may take years to detect a significant treatment effect. High-resolution computerized tomography is proposed as a more sensitive alternative, but this methodology may also require an extended period of time to determine a positive outcome (30, 31).

While a number of inflammatory mediators have been proposed as biomarkers for pulmonary emphysema, free DID crosslinks may have greater specificity for this disease because they are better indicators of alveolar wall changes responsible for airspace enlargement (32). Despite the possible effect of co-existing diseases on specificity, free DID may nevertheless play an important role in clinical trials, where significant differences in crosslink levels between closely matched experimental and control groups would provide strong evidence of therapeutic efficacy. The use of sputum and possibly breath condensate for measurement of free DID would also increase the specificity for pulmonary emphysema. However, the acceptance of free DID as a biomarker for pulmonary emphysema may ultimately depend on developing an accurate and reproducible method of analysis. There is no commonly accepted protocol for measuring DID, and the cost of expertise and equipment may be a rate-limiting factor in the widespread adoption of the biomarker (33).

To determine the role of free DID in evaluating drug efficacy, our laboratory incorporated this biomarker in a 28-day clinical trial of hyaluronan (HA), a long-chain polysaccharide, in patients with alpha-1 antiprotease deficiency induced pulmonary emphysema (34). Inhalation of aerosolized HA significantly decreased the amount of free DID in urine over a 28-day period following initiation of treatment, whereas levels in the placebo group remained unchanged. Free urinary DID was also a more sensitive indicator of a treatment effect than total DID in either urine or plasma. These results provide proof of concept for the use of free DID as a biomarker for therapeutic efficacy and suggest that inhaled HA may slow the progression of pulmonary emphysema. The potential therapeutic effects of this agent are further supported by studies showing that inhalation of HA mitigated the loss of pulmonary function in patients with acute exacerbations of COPD and prevented bronchoconstriction in those with exercise-induced asthma (35, 36).

The use of HA to treat pulmonary emphysema is based on previous studies demonstrating that pretreatment of the lung with hyaluronidase enhances elastase-induced airspace enlargement, while inhalation of exogenous HA has the opposite effect, preventing alveolar wall injury by binding to elastic fibers and protecting them from enzymatic degradation (37–41). The hydrophilic properties of HA may also increase the storage of energy in elastin, thereby mitigating the mechanical strain that contributes to airspace enlargement. This hypothesis is supported by a recent study indicating that HA and other proteoglycans reduce uneven distribution of forces in the extracellular matrix (42).

These findings provide a rationale for developing drugs that inhibit the broader process of disease emergence rather than individual components of the inflammatory reaction associated with airspace enlargement. Aside from alpha_1_-antiprotease augmentation in a small subgroup with genetically induced pulmonary emphysema, elastase inhibitors and other anti-inflammatory agents have shown only limited success in treating the disease (43–46). This may be due to the complexity of emergent phenomena, where reorganization of a system depends on numerous interactions at different levels of scale rather than the elementary properties of the constituents. Consequently, the loss of specific molecular components of lung injury could be circumvented by the higher-level effects of structural alterations in alveolar walls.

## Conclusion

Pulmonary emphysema involves multiple pathogenic mechanisms such as excess elastase activity, oxidative stress, and alpha-1 antiprotease deficiency. The interaction of these various processes may lead to converging patterns of injury, including loss of elastin crosslinks, microscopic fragmentation of elastic fibers, and macroscopic rupture of alveolar walls. These events at different levels of scale are consistent with an emergent phenomenon, suggesting that effective treatment of pulmonary emphysema may depend on the use of therapeutic agents prior to a phase transition involving alveolar wall rupture. While no accepted method for accurately measuring the early stages of pulmonary emphysema is currently available, the use of free DID to detect initial changes in airspace size may permit timely therapeutic intervention that reduces the risk of respiratory failure.

## Ethics statement

The studies involving humans were approved by the institutional review boards at both Montefiore Medical Center and St John’s University under protocol IRB-FY2021-184. The studies were conducted in accordance with the local legislation and institutional requirements. The human samples used in this study were acquired from gifted from another research group. Written informed consent for participation was not required from the participants or the participants’ legal guardians/next of kin in accordance with the national legislation and institutional requirements. The animal study was approved by the institutional review board at St John’s University under protocol 1915. The study was conducted in accordance with the local legislation and institutional requirements.

## Author contributions

JC: Conceptualization, Writing – original draft, Writing – review & editing.
